# Supplementary material for: Bipartite genome and structural organization of the parvovirus Acheta domesticus segmented densovirus
Source: Nat Commun. 2023 Jun 14;14:3515. doi: 10.1038/s41467-023-38875-x (PMC10267136; doi:10.1038/s41467-023-38875-x)
Supplement: Supplementary file 1 — Supplementary Information [file 41467_2023_38875_MOESM1_ESM.pdf]

S1 Table: The exact positions of the open reading frames (ORFs) located on the ns and vp segments of Acheta domesticus segmented densovirus, respectively.

ns segment

ORF name	Start position	End position
ORF1 (NS1)	471 nt	2858 nt
ORF2 (NS2)	559 nt	1695 nt
ORF3	1756 nt	1824 nt
ORF4 (NS1-N1 2nd exon) [a]	2177 nt	2209 nt
ORF5 (NS1-N2 2nd exon) [a]	2351 nt	2500 nt

[a]ORF2 has no ATG start codon

vp segment

ORF name	Start position	End position
vpORF1	483 nt	1562 nt
vpORF2	1543 nt	1854 nt
vpORF3	1859 nt	2899 nt

S2 Table: Intron and exon boundaries of the Acheta domesticus segmented densovirus spliced transcripts

Transcript exon	Start pos.	End pos.	Transcript intron	Start pos.	End pos.
**ns segment**					
Transcript 2, exon 1	579 nt	1391 nt	Transcript 2, intron 1	1392 nt	2185 nt
Transcript 2, exon 2	2186 nt	2209 nt			
Transcript3, exon 1	579 nt	1818 nt	Transcript 3, intron 1	1819 nt	2423 nt
Transcript 3, exon 2	2424 nt	2500 nt			
Transcript5, exon 1	949 nt	1391 nt	Transcript 5, intron 1	1392 nt	2185 nt
Transcript 5, exon 2	2186 nt	2858 nt			
Transcript6, exon 1	1756 nt	1818 nt	Transcript 6, intron 1	1819 nt	2423 nt
Transcript 6, exon 2	2424 nt	2858 nt			
**vp segment**					
Transcript 3, exon 1	1543 nt	1752 nt	Transcript 3, intron 1	1753 nt	2032 nt
Transcript 3, exon 2	2033 nt	2899 nt			
Transcript 4, exon 1	1543 nt	1752 nt	Transcript 4, intron 1	1753 nt	2611 nt
Transcript 4, exon 2	2612 nt	2899 nt			

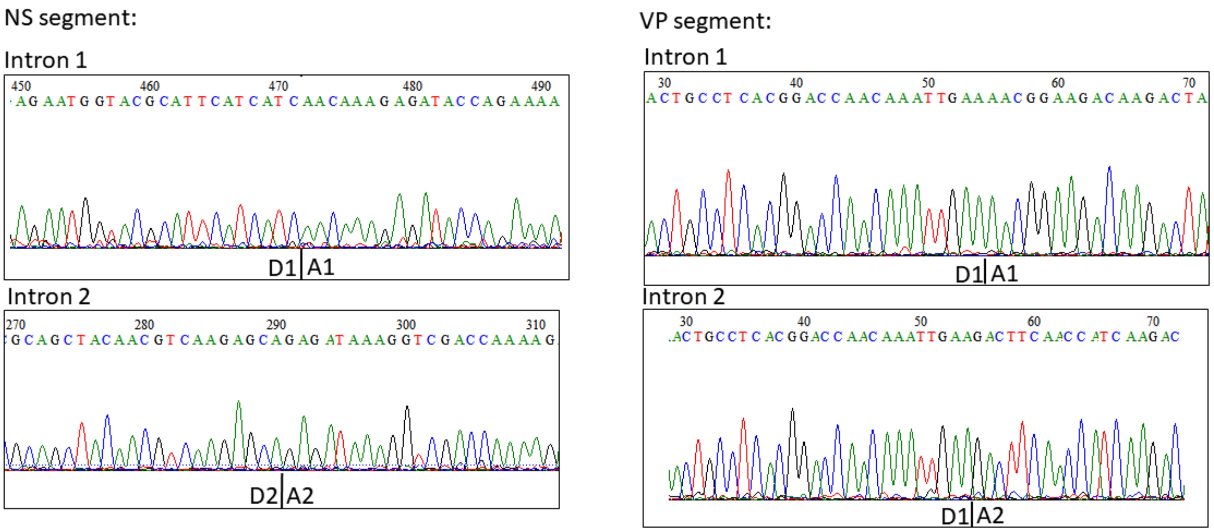

S3 Figure: Electrophoretograms showing the spliced-out introns of sequenced cDNA of mRNA derived from AdSDV-infected common house crickets.

S4 Figure: Protein sequencing results of the nano-liquid chromatography tandem mass spectrometry, searched against the complete translated Acheta domesticus segmented densovirus (AdSDV) genome as well as the NCBI non-redundant protein sequence database.

55 kDa, EC band

Protein FDR Confidence: Sequest HT	Description	Coverage [%]	# Peptides	# PSMs	# Unique Peptides	# AAs	MW [kDa]	calc. pI	Score Sequest HT: Sequest HT	# Peptides
High	ORF1 [AdSDV]	64	25	159	25	377	43	6.74	331.01	25
High	E280Q C383AC [Ecoli]	53	22	103	22	393	44.6	5.88	204.73	22
High	Keratin K1C10 [Human]	44	22	90	22	593	59.5	5.21	185.78	22
High	Albumin [Bovine]	15	8	19	8	607	69.2	6.18	51.07	8

50 kDa, EC band

Protein FDR Confidence: Sequest HT	Description	Coverage [%]	# Peptides	# PSMs	# Unique Peptides	# AAs	MW [kDa]	calc. pI	Score Sequest HT: Sequest HT	# Peptides
High	ORF1 [AdSDV]	66	28	195	28	377	43	6.74	364.79	28
High	Keratin K1C10 [Human]	42	16	47	16	593	59.5	5.21	72.27	16

43 kDa, EC band

Protein FDR Confidence: Sequest HT	Description	Coverage [%]	# Peptides	# PSMs	# Unique Peptides	# AAs	MW [kDa]	calc. pI	Score Sequest HT: Sequest HT	# Peptides
High	ORF1 [AdSDV]	80	38	1909	38	377	43	6.74	4305.85	38
High	Keratin K1C10 [Human]	34	14	43	14	593	59.5	5.21	80.88	14

55 kDa, FC band

Protein FDR Confidence: Sequest HT	Description	Coverage [%]	# Peptides	# PSMs	# Unique Peptides	# AAs	MW [kDa]	calc. pI	Score Sequest HT: Sequest HT	# Peptides
High	Keratin K1C10 [Human]	42	20	86	20	593	59.5	5.21	160.94	20
High	ORF1 [AdSDV]	63	21	63	21	377	43	6.74	78.44	21
High	OxDC C383A [Ecoli]	33	10	16	10	393	44.6	5.8	13	10
High	Albumin [Bovine]	6	3	4	3	607	69.2	6.18	5.45	3

50 kDa, FC band

Protein FDR Confidence: Sequest HT	Description	Coverage [%]	# Peptides	# PSMs	# Unique Peptides	# AAs	MW [kDa]	calc. pI	Score Sequest HT: Sequest HT	# Peptides
High	ORF1 [AdSDV]	63	21	59	21	377	43	6.74	92.93	21
High	Keratin K1C10 [Human]	37	14	40	14	593	59.5	5.21	71.58	14

43 kDa, FC band

Protein FDR Confidence: Sequest HT	Description	Coverage [%]	# Peptides	# PSMs	# Unique Peptides	# AAs	MW [kDa]	calc. pI	Score Sequest HT: Sequest HT	# Peptides
High	ORF1 [AdSDV]	72	32	698	32	377	43	6.74	1404.73	32
High	Keratin K1C10 [Human]	41	19	50	19	593	59.5	5.21	92.72	19

38 kDa, FC band

Protein FDR Confidence: Sequest HT	Description	Coverage [%]	# Peptides	# PSMs	# Unique Peptides	# AAs	MW [kDa]	calc. pI	Score Sequest HT: Sequest HT	# Peptides
High	ORF1 [AdSDV]	66	30	640	30	377	43	6.74	1375.34	30
High	Keratin K1C10 [Human]	34	18	46	18	593	59.5	5.21	84.43	18
High	OxDC C383A [Ecoli]	7	1	2	1	393	44.6	5.8	3.51	1

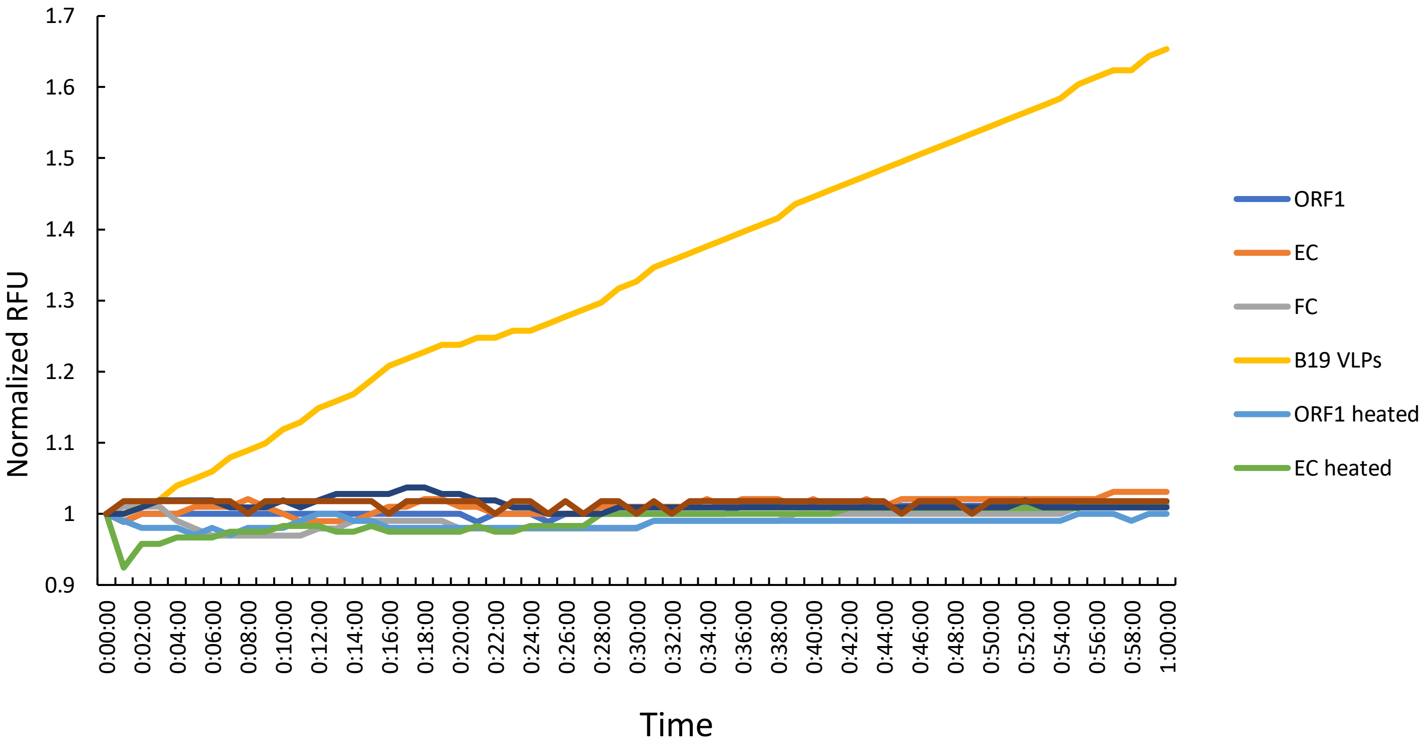

S5 Figure: Colorimetric assay measuring the PLA2 activity of the Acheta domesticus segmented densovirus (AdSDV) capsids, using parvovirus B19 virus like particles (VLPs) as positive control. Values are normalized to the ones measured at the first timepoint of the assay. EC abbreviates empty capsids pulled from the 20% band of the sucrose step gradient, used for AdSDV purification. FC capsids derive from the 30% band of the sucrose step gradient. ORF1 indicates VLPs assembled exclusively from the protein product of vp ORF1, lacking the PLA2 domain, and functions as negative control. Purified particles were heated to 60°C to obtain the data indicated as "heated". Time is shown in the hour:minute:second format. Source data to this figure are provided as a Source Data file. N=2 quasi-independent experiments (virus populations from which DNA was extracted derive from three purification event of the same batch of AdSDV-infected common house crickets).

A

**Processing and Refinement Parameters**	FC	EC1	EC2	EC	ORF1
Total number of micrographs	1411	970	1002	1127	1700
Reconstruction software	cisTEM	cisTEM	cisTEM	cisTEM	cisTEM
Defocus range (µm)	0.65-4.00	0.81-4.00	0.8-4.32	0.62-4.41	0.57-2.57
Electron dose ($e^{-}/Å^2$)	75	60	60	75	60
Frames/micrograph	50	50	50	50	50
Pixel size (Å/pixel)	1.041	1.038	1.038	1.041	1.049
Starting number of particles	188074	46082	58680	98920	18598
Particles used for final map	150469	30065	48354	59211	13019
B-factor used for final map ($Å^2$)	20 (Post-Cut-Off B-factor)	10 (Post-Cut-Off B-factor)	10 (Post-Cut-Off B-factor)	20 (Post-Cut-Off B-factor)	20 (Post-Cut-Off B-factor)
Resolution of final map (Å)	2.3	3	3.1	2.5	3.3
Residue range (VP1)	47-377	49-366	49-366	49-366	49-366
Map correlation coefficient	0.6528	0.629	0.6115	0.7147	0.6774
RMSD (root-mean-square deviation) [bonds] (Å)	0.010	0.008	0.011	0.009	0.011
RMSD [angles] (Å)	0.852	0.788	0.863	0.772	0.849
All-atom clash score	12.19	9.35	12.7	15.3	13.6
Favored (%)	95	98.1	97.5	96.1	95.9
Allowed (%)	4.5	1.6	2.5	3.6	3.8
Outliers (%)	0.5	0.3	0	0.3	0.3
Rotamer outliers (%)	0	0.3	0	0	0
C-β deviations	0	0	0	0	0

B

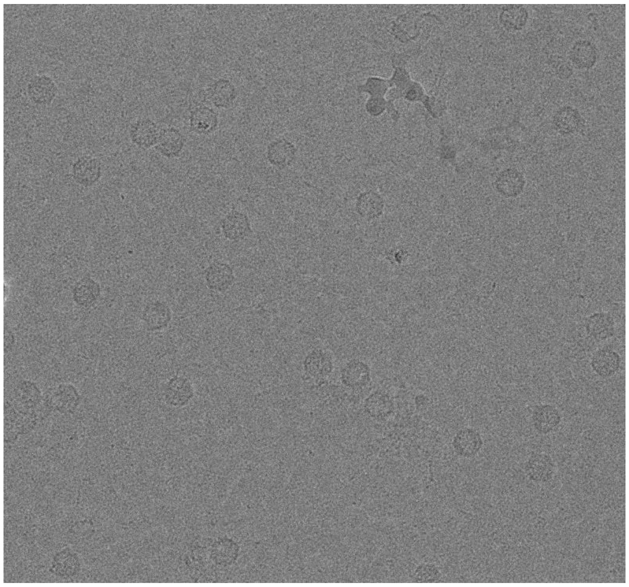

EC1

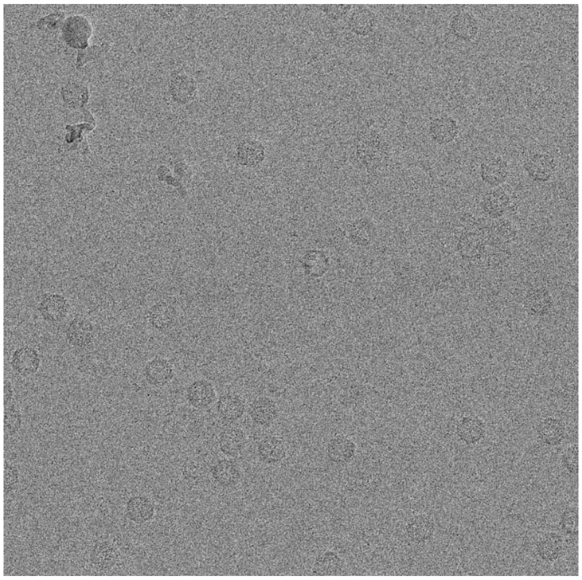

EC2

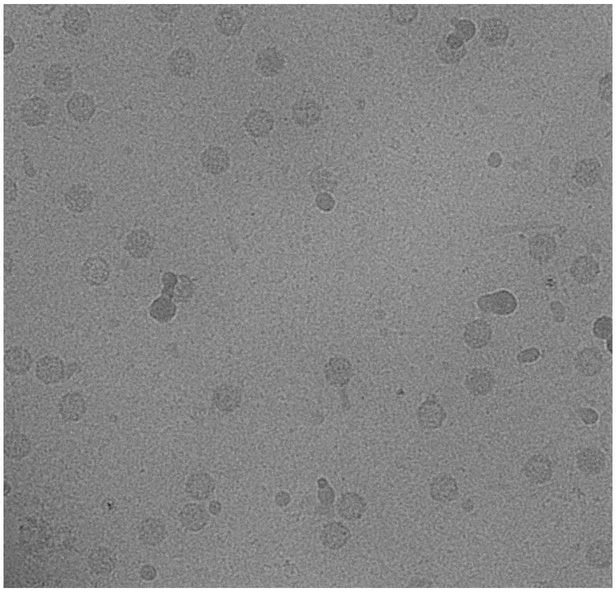

EC

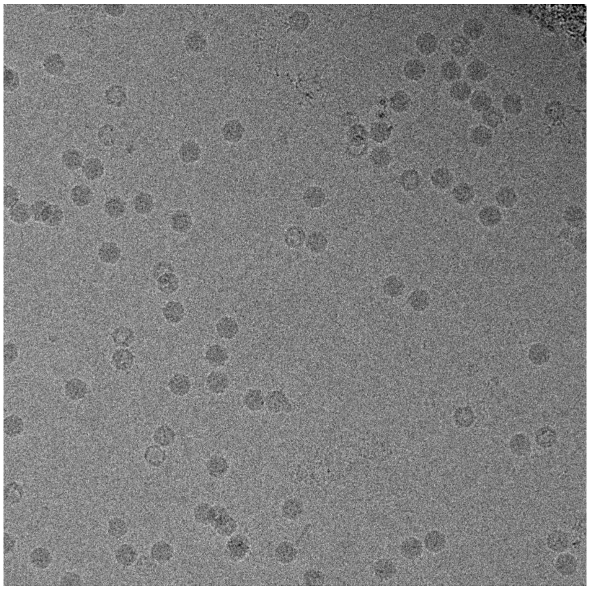

FC

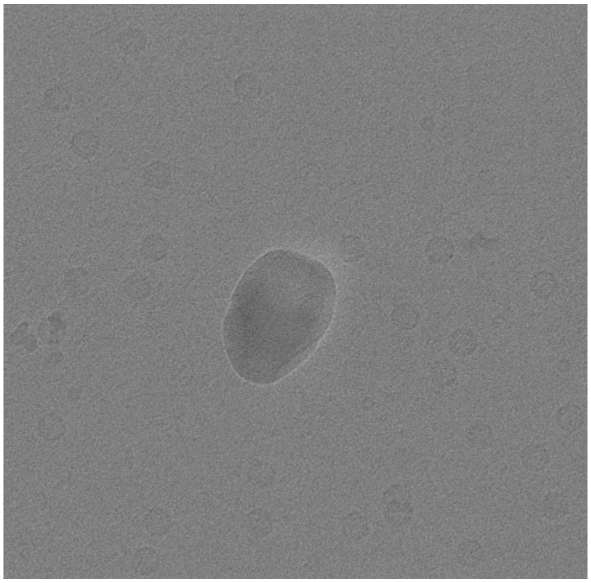

ORF1 VLPs

S6 Figure: Data collection, refinement statistics and example micrographs. (A) The table shows the data collection parameters and refinement statistics of the Acheta domesticus segmented densovirus capsids and vpORF1 only virus-like particles (VLPs). (B) Example raw micrographs of each data collection. Abbreviations: EC – empty capsids, FC – Full capsids, ORF – open reading frame, VLPs – Virus-like particles.

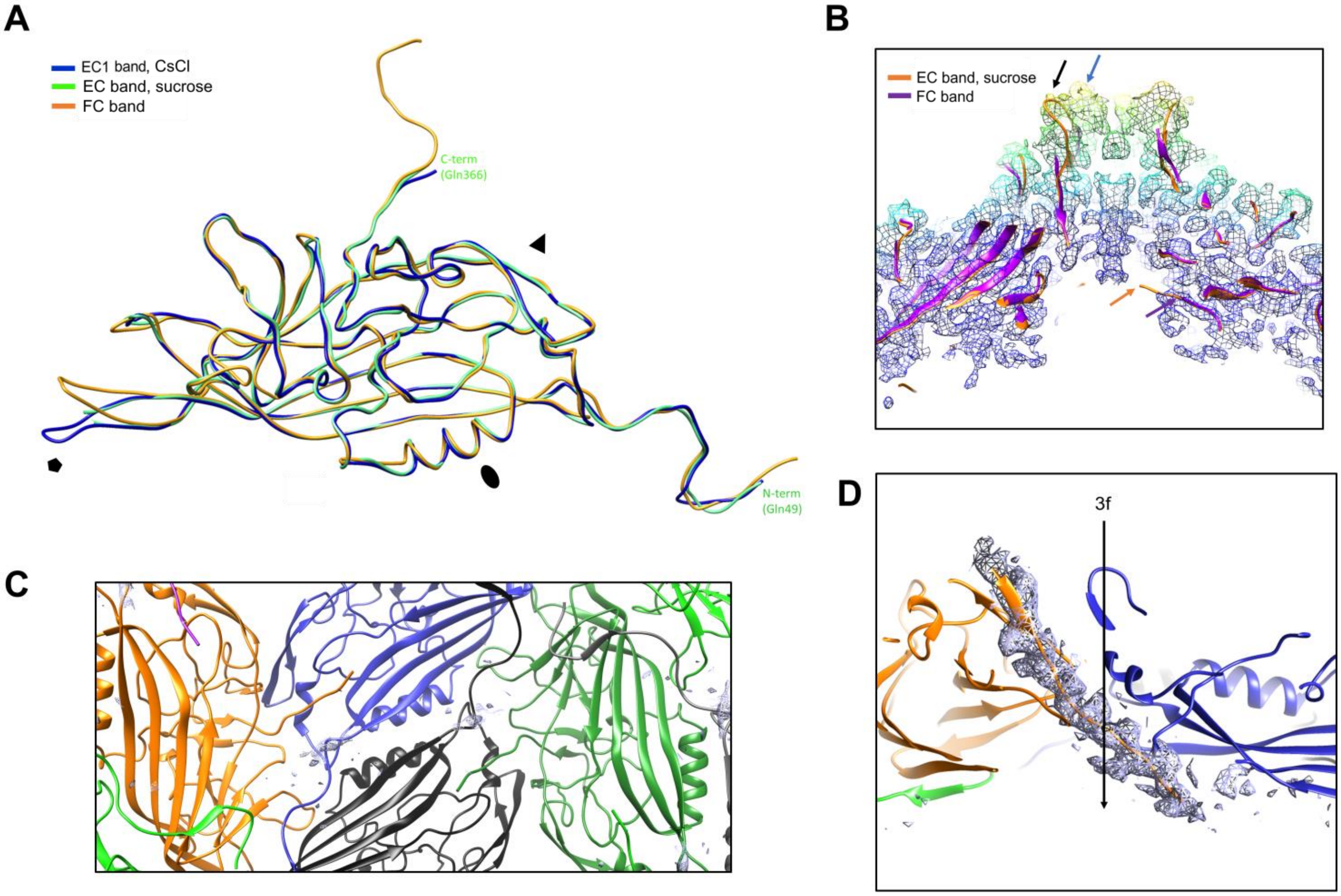

S7 Figure: The EC band, pulled from the sucrose step gradient, contains a mixed population on Acheta domesticus segmented densovirus (AdSDV) capsids. (A) When superimposing the structural model of the EC band monomer with those of the EC bands pulled from the continuous CsCl gradient and with the (FC) capsid monomers, the EC sucrose capsids display the disordered N- and C termini of the CsCl EC capsids, accompanied by a disordered DE loop, as result of the conformation difference between the EC and EC1-2 populations. (B) Cross section of the EC sucrose capsid fivefold axis, colored radially, with the electron density indicated by the colored mash (σ=1). The model built into the density is shown as ribbon diagrams, superimposed with that of the FC capsids. The EC sucrose capsids display the DE loop density characteristic for both the FC fivefold axis (black arrow) and the CsCl EC1 and 2 fivefold axes (blue arrow). Note the density-filled fivefold channel, which lacks the connection to the first N-terminally ordered residue (orange arrow for FC, magenta arrow for EC sucrose capsids), unlike in case of the EC capsids. (C) The ssDNA-binding luminal region, located directly under the twofold symmetry axis, shown as ribbon diagrams. Display of the electron density map was zoned to the nucleic acid models exclusively, indicating the lack of ordered nucleotides (σ=1). (D) Side view of the EC sucrose capsid threefold annulus, with the black arrow indicating the location of the threefold axis. The density, displayed as the purple mash and zoned on the final C-terminal 20 residues, indicates that the ordered segment ends under the threefold axis, leaving the final eleven residues disordered, similarly to the EC1, EC2 and vpORF1 only capsids.

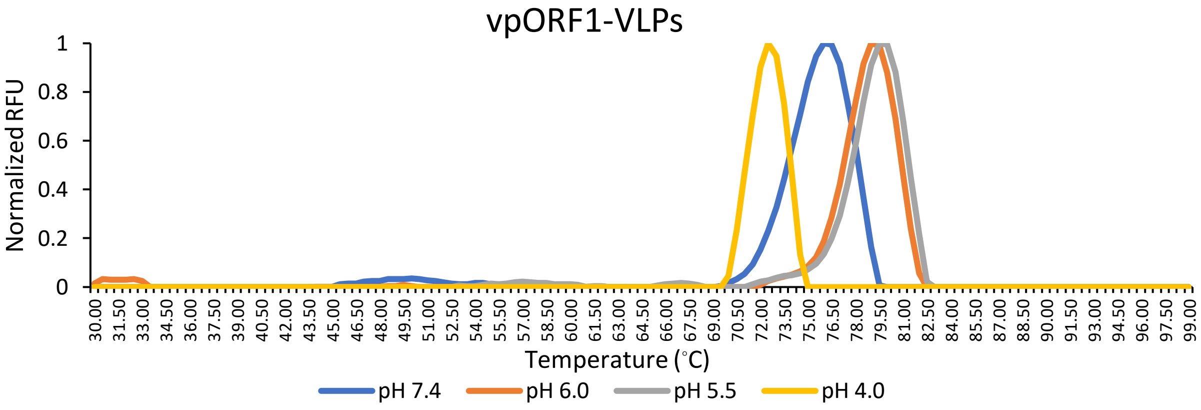
vpORF1-VLPs
Normalized RFU
0 0.2 0.4 0.6 0.8 1
30.00 31.50 33.00 34.50 36.00 37.50 39.00 40.50 42.00 43.50 45.00 46.50 48.00 49.50 51.00 52.50 54.00 55.50 57.00 58.50 60.00 61.50 63.00 64.50 66.00 67.50 69.00 70.50 72.00 73.50 75.00 76.50 78.00 79.50 81.00 82.50 84.00 85.50 87.00 88.50 90.00 91.50 93.00 94.50 96.00 97.50 99.00
Temperature (°C)
pH 7.4
pH 6.0
pH 5.5
pH 4.0

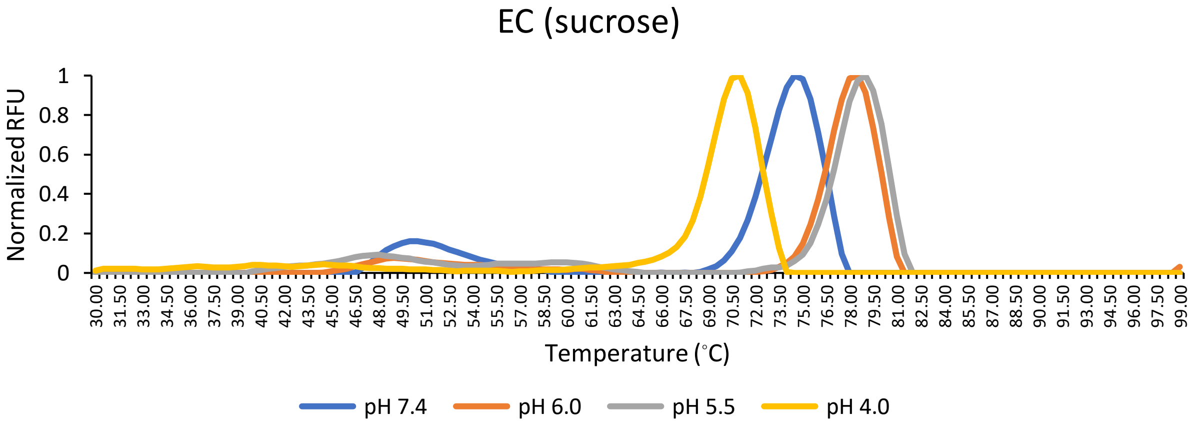
EC (sucrose)
Normalized RFU
0 0.2 0.4 0.6 0.8 1
30.00 31.50 33.00 34.50 36.00 37.50 39.00 40.50 42.00 43.50 45.00 46.50 48.00 49.50 51.00 52.50 54.00 55.50 57.00 58.50 60.00 61.50 63.00 64.50 66.00 67.50 69.00 70.50 72.00 73.50 75.00 76.50 78.00 79.50 81.00 82.50 84.00 85.50 87.00 88.50 90.00 91.50 93.00 94.50 96.00 97.50 99.00
Temperature (°C)
pH 7.4
pH 6.0
pH 5.5
pH 4.0

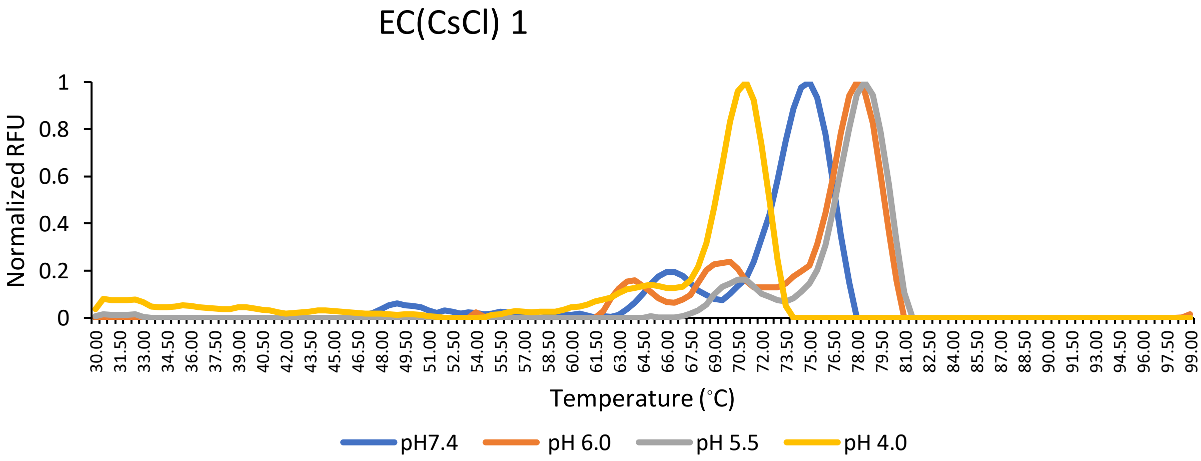
EC(CsCl) 1
Normalized RFU
0 0.2 0.4 0.6 0.8 1
30.00 31.50 33.00 34.50 36.00 37.50 39.00 40.50 42.00 43.50 45.00 46.50 48.00 49.50 51.00 52.50 54.00 55.50 57.00 58.50 60.00 61.50 63.00 64.50 66.00 67.50 69.00 70.50 72.00 73.50 75.00 76.50 78.00 79.50 81.00 82.50 84.00 85.50 87.00 88.50 90.00 91.50 93.00 94.50 96.00 97.50 99.00
Temperature (°C)
pH7.4
pH 6.0
pH 5.5
pH 4.0

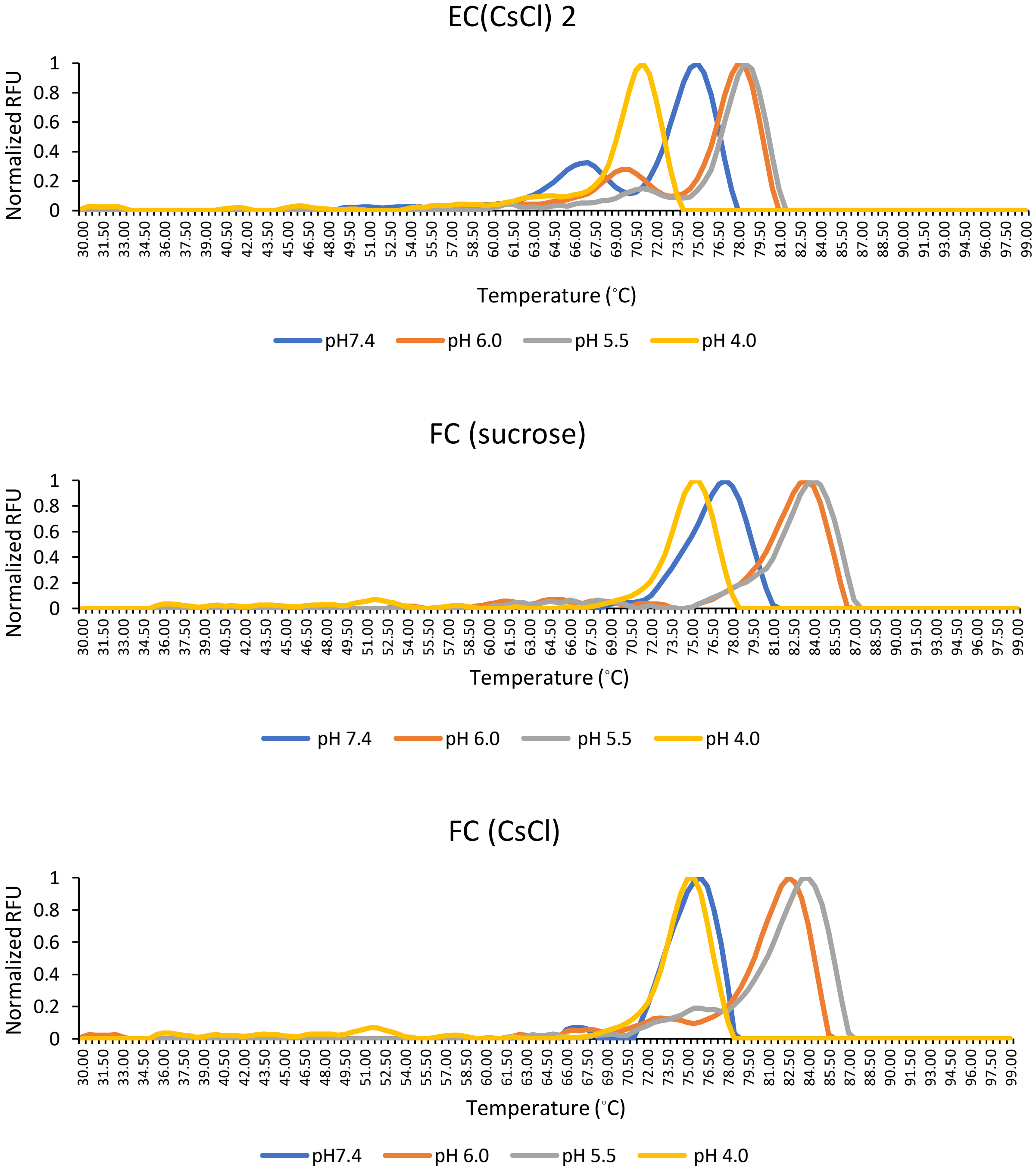

S8 Figure: Differential scanning fluorometry (DSF) profiles of all Acheta domesticus segmented densovirus capsid fractions. The obtained curves were normalized to the maximum values of each run, respectively. Values 0> are presented as 0. FC abbreviates full capsids pulled either from the sucrose or CsCl gradient, while EC stands for empty capsid fractions. VLPs abbreviate virus-like particles. Source data to this figure are provided as a Source Data file.
