# Supplementary material for: Bipartite genome and structural organization of the parvovirus Acheta domesticus segmented densovirus
Source: Nat Commun. 2023 Jun 14;14:3515. doi: 10.1038/s41467-023-38875-x (PMC10267136; doi:10.1038/s41467-023-38875-x)
Supplement: Supplementary file 3 — Reporting Summary [file 41467_2023_38875_MOESM3_ESM.pdf]

nature portfolio

Corresponding author(s): Judit J. Penzes

Last updated by author(s): Nov 14, 2022

Reporting Summary

Nature Portfolio wishes to improve the reproducibility of the work that we publish. This form provides structure for consistency and transparency in reporting. For further information on Nature Portfolio policies, see our Editorial Policies and the Editorial Policy Checklist.

Statistics

For all statistical analyses, confirm that the following items are present in the figure legend, table legend, main text, or Methods section.

n/a	Confirmed	
☐	☒	The exact sample size (*n*) for each experimental group/condition, given as a discrete number and unit of measurement
☒	☐	A statement on whether measurements were taken from distinct samples or whether the same sample was measured repeatedly
☒	☐	The statistical test(s) used AND whether they are one- or two-sided<br>*Only common tests should be described solely by name; describe more complex techniques in the Methods section.*
☒	☐	A description of all covariates tested
☒	☐	A description of any assumptions or corrections, such as tests of normality and adjustment for multiple comparisons
☐	☒	A full description of the statistical parameters including central tendency (e.g. means) or other basic estimates (e.g. regression coefficient) AND variation (e.g. standard deviation) or associated estimates of uncertainty (e.g. confidence intervals)
☒	☐	For null hypothesis testing, the test statistic (e.g. *F*, *t*, *r*) with confidence intervals, effect sizes, degrees of freedom and *P* value noted<br>*Give P values as exact values whenever suitable.*
☒	☐	For Bayesian analysis, information on the choice of priors and Markov chain Monte Carlo settings
☒	☐	For hierarchical and complex designs, identification of the appropriate level for tests and full reporting of outcomes
☒	☐	Estimates of effect sizes (e.g. Cohen's *d*, Pearson's *r*), indicating how they were calculated

Our web collection on statistics for biologists contains articles on many of the points above.

Software and code

Policy information about availability of computer code

Data collection	CryoEM micrographs were collected by Leginon 3.6 (http://leginon.org)
Data analysis	Figures were made by UCSF Chimera v1.13.1 (https://www.cgl.ucsf.edu/chimera/) and PYMOL v2.3.4 (https://pymol.org/2/)<br>High resolution data collection was carried out by Leginon v2.0 and v3.2 (http:/nramm.nysbc.org)<br>Correcting beam-induced motion by UCSF MotionCor2 v1.4.2 (https://msg.ucsf.edu/software)<br>Single particle reconstruction was carried out by CisTEM (https://cistem.org/)<br>Electron density map conversion: MAPMAN v3.6 (http:/mapman.gabipd.org)<br>Tools used for modeling and refining the electron density obtained by the CryoEM data:<br>Coot (https://www2.mrc-lmb.cam.ac.uk/personal/pemsley/coot/)<br>PHENIX (https://phenix-online.org/)<br>Phylogenetic calculations:<br>- Alignment editing: Unipro Ugene v44.0 (http://ugene.net/)<br>- Preparing the alignment: T-coffee Expresso in PDB mode (https://tcoffee.org/)<br>- Model selection: prottest v2.4 (https://github.com/ddarriba/prottest3)<br>- executing phylogeny calculations: BEAST v1.10.4 (https://beast.community/)<br>- tree visualization: FigTree v1.4.4 (https://beast.community/)<br>Mass spectrometry:<br>- obtained mass spectra were analyzed and searched by Proteome Discoverer 2.4 (https://www.thermofisher.com/us/en/home/industrial/mass-spectrometry/liquid-chromatography-mass-spectrometry-lc-ms/lc-ms-software/multi-omics-data-analysis/proteome-discoverer-software.html)

Genome assembly:
- Staden package v2.0.0 and v4.11.2 (https://staden.sourceforge.net/)

For manuscripts utilizing custom algorithms or software that are central to the research but not yet described in published literature, software must be made available to editors and reviewers. We strongly encourage code deposition in a community repository (e.g. GitHub). See the Nature Portfolio guidelines for submitting code & software for further information.

Data

Policy information about availability of data

All manuscripts must include a data availability statement. This statement should provide the following information, where applicable:

- Accession codes, unique identifiers, or web links for publicly available datasets
- A description of any restrictions on data availability
- For clinical datasets or third party data, please ensure that the statement adheres to our policy

Nucleotide sequences are available under the GenBank Accession numbers of OP436269 (NS segment) and OP436270 (VP segment).
Protein structures were deposited into the RCSB Protein Data Bank under ID numbers 8ER8, 8ERK, 8EU5, 8EU6, 8EU7
Protein sequences were submitted to the NCBI non-redundant protein database under accession numbers WDB01642, WDB01643, WDB01644, WDB01645 and WDB01646.
Density maps generated via cryo-electron microscopy and 3D image reconstruction were deposited to the Electron Microscopy Data bank, under identification numbers EMD-28604, EMD-28550, EMD-28553, EMD-28605 and EMD-28607.
Databases used:
RCSB Protein Data Bank
NCBI non-redundant protein database

Human research participants

Policy information about studies involving human research participants and Sex and Gender in Research.

Reporting on sex and gender	N/A
Population characteristics	N/A
Recruitment	N/A
Ethics oversight	N/A

Note that full information on the approval of the study protocol must also be provided in the manuscript.

Field-specific reporting

Please select the one below that is the best fit for your research. If you are not sure, read the appropriate sections before making your selection.

☐ Life sciences ☐ Behavioural & social sciences ☒ Ecological, evolutionary & environmental sciences

For a reference copy of the document with all sections, see nature.com/documents/nr-reporting-summary-flat.pdf

Ecological, evolutionary & environmental sciences study design

All studies must disclose on these points even when the disclosure is negative.

Study description

Common house crickets (Acheta domesticus) on a Canadian cricket rearing farm exhibited signs and death suggesting ongoing viral infection. The infectious agent was identified by blunt-end cloning isolated DNA and subsequent sequencing of the viral genome, revealing a new densovirus with a segmented genome. Healthy house crickets were subsequently infected by inoculation of purified virus, either by feeding or by direct fat body injections, confirming the new Acheta domesticus segmented densovirus (AdSDV) as the causative agent of the outbreak. We resolved and analyzed the transcription strategy of AdSDV by isolating mRNA directly from the infected animals. We purified AdSDV virus particles directly from infected house crickets as well as recombinantly expressed self-assembling virus like particles using the Bac-to-Bac expression system. The capsid structure of all purified infectious, non-infectious and recombinantly expressed particles was resolved.

Research sample

Deceased common house crickets (A. domesticus) sent directly from the rearing facility (approx. 100 individuals), as these animals displayed the symptoms first of the densovirus detected in this study.
Alive, healthy common house crickets, free from previously known densovirus infection (approx. 600 individuals)
- age: nymphs of the final instar, wingless, 10-15 mm
- rational: the aim was to obtain at least 5 mg purified virus to carry out structural studies and to have enough particles for further experiments in case the virus purification and structural studies did not work out. We agreed on the numbers we used based on initial virus yield of the insects obtained from the farm, where the break-out was first recognized.
- justification of age: the vast majority of the initial deceased samples from the farm had trouble with developing into adulthood. The

juveniles still had to be large enough for direct fat body injections, for which purpose the last instar appeared to be the most suitable choice.

Sampling strategy	The initial sample size obtained from the farm was a collection of deceased nymphs, at various stages.<br>The aim was to obtain at least 5 mg purified virus to carry out structural studies and to have enough particles for further experiments in case the virus purification and structural studies did not work out. We agreed on the numbers we used based on initial virus yield of the insects obtained from the farm, where the break-out was first recognized. Consequently, the sampling strategy represents the individuals most susceptible for the virus infection.
Data collection	Collecting deceased animals: carried out at the farm, which would like to remain anonymous. Approx. 100 deceased animals, previously exhibiting the signs of infection, were picked up and placed into a sterile container. The tightly closed container was mailed to our laboratory.<br>They were subjected to virus purification by Hanh T Pahm. Instruments used: blender, Beckman-Coulter bench top centrifuge, Beckman-Coulter Class S ultracentrifuge, 60ti ultracentrifuge rotor.<br>The purified virus was subjected to DNA isolation and molecular cloning:<br>- Instruments used: BioRad PCR machine, microcentrifuge, micropipets, 30-37 Celsius incubator.<br>Infecting healthy house crickets:<br>- By mouth: 200 ul of AdSDV suspension in phosphate buffered saline in 1 mg/ml concentration was mixed to dry commercially available cricket food. The mixture was served on a sterile Parafilm layer to the animals. Data was recorded into a notebook by pen and paper by Hanh T. Pham and Judit J. Penzes.<br>- By injections: 2 ul of the above-mentioned suspension was introduced directly into the abdominal fat body of the living individuals, using a delicate 1 ml syringe with an "insuline needle". Data was recorded into a notebook by pen and paper by Judit J. Penzes.<br>Once all crickets displayed symptoms they were subjected to:<br>- Total RNA purification by Judit J. Penzes<br>Instruments used: blender, Eppendorf microcentrifuge, micropipets, BioRad PCR machine, heated waterbath<br>- Virus purification by Judit J. Penzes<br>Instruments used: belnder, Beckman-Coulter bench top centrifuge, Beckman-Coulter Class S ultracentrifuge, 60ti and SW41 ultracentrifuge rotors.<br>Purified virus was subjected to:<br>- High resolution data collection by Emmanuel W. Smith and Paul Chipman<br>Instruments used: Mark IV Vitrobot, dewar, clipping station, FEI Titan Krios electron microscope<br>- qPCR by Judit J. Penzes<br>Instruments used: Eppendorf microcentrifuge, micropipets, Bio Rad CFX96 qPCR machine
Timing and spatial scale	2014, August: collecting deceased insects<br>2015, September to 2016, June: experimentally infecting insects by the above-described methods to study transmission and host spectrum of the virus<br>2016, June to 2017, August: infecting insects for mRNA isolation<br>2020, May to 2021, July: infecting insects for virus purification<br>2019, August: recombinantly expressing virus particles using the Sf9-mediated Bac-toBac expression system<br>2020, September to 2021, June: Collecting cryoEM data
Data exclusions	Crickets, which died within three days of viral inoculation were discarded as their death could not be attributed to viral infection.
Reproducibility	qPCR studies: Performed successfully on two different occasions in triplicates. Virus derived from the same batch of infected crickets each time.<br>All PLA2 activity assay reactions were performed twice, on the same quasi-independent virus samples; samples derived from two separate virus purification events, but from the same batch of infected house crickets.<br>All differential scanning fluorimetric assay reactions were done in duplicates and repeated twice on the same quasi-independent batches, mentioned above.<br>All attempts at replication in all experiments were successful.
Randomization	House crickets were randomly selected to be inoculated either by virus suspension or by sterile PBS. The insects were placed in two opaque plastic containers, which only had a small enough opening, so that one can individually grab them. They were devided simply by emptying the large canvas bag in two equal halves into each container. To ensure that they were healthy, they had to jump over into the container themselves. First, the ones to be treated by PBS were individually picked from one container. To ensure that individuals were picked, which had not been stressed out, we picked the ones to be treated with the virus from the second container.
Blinding	As only one person executed all the experimental infection studies, blinding was not possible. In the other aspects of the study blindig has no relevance as these were experiments of molecular and structural biology, directly targeting the virus itself or its components. Rationale: at this point all animals of the entire treatment group was poured into a blender, hence they ceased to be evaluated as individual animals, as they all blended together.

Did the study involve field work? ☐ Yes ☒ No

Reporting for specific materials, systems and methods

We require information from authors about some types of materials, experimental systems and methods used in many studies. Here, indicate whether each material, system or method listed is relevant to your study. If you are not sure if a list item applies to your research, read the appropriate section before selecting a response.

Materials & experimental systems

n/a	Involved in the study
☒	☐ Antibodies
☐	☒ Eukaryotic cell lines
☒	☐ Palaeontology and archaeology
☐	☒ Animals and other organisms
☒	☐ Clinical data
☒	☐ Dual use research of concern

Methods

n/a	Involved in the study
☒	☐ ChIP-seq
☒	☐ Flow cytometry
☒	☐ MRI-based neuroimaging

Eukaryotic cell lines

Policy information about cell lines and Sex and Gender in Research

Cell line source(s)	American Type Culture Collection (ATCC)
Authentication	Directly ordered from ATCC as Sf9 cells of CRL-1711 clone number. The cell line was not authenticated by us, but as it was ordered from the type culture collection directly, displayed the expected morphology and produced high amount of virus-like particles upon transfected with recombinant baculovirus DNA, we believe this step could be omitted.
Mycoplasma contamination	There could be no Mycoplasma contamination detected.
Commonly misidentified lines (See ICLAC register)	Sf21

Animals and other research organisms

Policy information about studies involving animals; ARRIVE guidelines recommended for reporting animal research, and Sex and Gender in Research

Laboratory animals	No vertebrates or higher invertebrates were involved in this study. Common house crickets (Acheta domesticus), Gryllus bimaculatus and Grylloides sigillatus were the only animal species involved, all orthopteran arthropods. All were in the last wingless nymph instar. It is difficult to tell the age of an insect when they arrive at this stage, as transition between instars depends on environmental and nutrition factors. These insects were all directly ordered from the rearing facility at the desired instar.
Wild animals	All animals were collected from farms. No wild animals were used in this study.
Reporting on sex	Both sexes were used in the study.
Field-collected samples	There were no field-collected samples used in this study.
Ethics oversight	Only insects were used in this study, which are not under such regulations.

Note that full information on the approval of the study protocol must also be provided in the manuscript.
